# Does Coenzyme Q10 Supplementation Improve Human Oocyte Quality?

**DOI:** 10.3390/ijms22179541

**Published:** 2021-09-02

**Authors:** Cristina Rodríguez-Varela, Elena Labarta

**Affiliations:** 1 IVI Foundation—IIS La Fe, 46026 Valencia, Spain; elena.labarta@ivirma.com; 2 IVIRMA Valencia, 46015 Valencia, Spain

**Keywords:** Coenzyme Q10, oocyte quality, mitochondrial function, antioxidant, ROS, ATP

## Abstract

Acquiring oocyte competence requires optimal mitochondrial function and adequate ATP levels. In this context, CoQ10 supplementation may improve human oocyte quality and subsequent reproductive performance given its role in ATP synthesis and mitochondrial protection from ROS oxidative damage. In infertility treatments, CoQ10 therapy can be orally supplied to promote a more favorable environment for oocyte development in vivo or by its addition to culture media in an attempt to improve its quality in vitro. Human clinical studies evaluating the impact of CoQ10 on reproductive performance are summarized in this review, although the available data do not clearly prove its ability to improve human oocyte quality. The main objective is to provide readers with a complete overview of this topic’s current status as well as the keys for potential future research lines that may help to take this therapy to clinical practice. Indeed, further clinical trials are needed to confirm these results along with molecular studies to evaluate the impact of CoQ10 supplementation on oxidative stress status and mitochondrial function in human gametes.

## 1. Introduction

**Figure 1:**
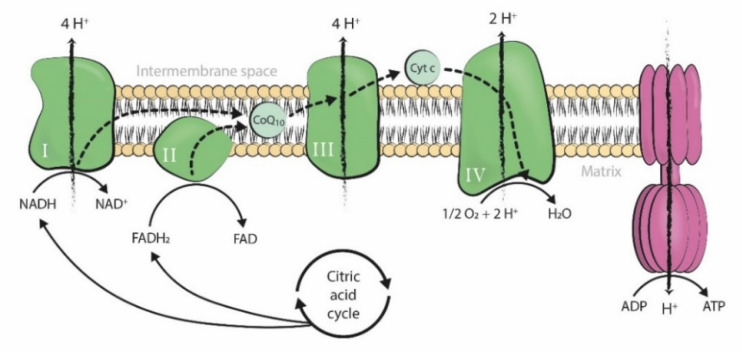
Mitochondrial respiratory chain (Complexes I, II, III, and IV; CoQ10, and cytochrome c) and the F1-F0 ATPase in the inner mitochondrial membrane. The movement of electrons throughout the mitochondrial respiratory chain is coupled with the transfer of protons across the membrane to the intermembrane space, generating an electrochemical proton gradient that is harnessed by F1-F0 ATPase to phosphorylate ADP into ATP (Figure from [2]). ADP: adenosine triphosphate. ATP: adenosine triphosphate. Pi: inorganic phosphate. H+: hydrogen ion (proton). NADH: nicotinamide adenine dinucleotide, reduced form. FADH2: flavin adenine dinucleotide, reduced form. NAD+: nicotinamide adenine dinucleotide, oxidized form. FAD: flavin adenine dinucleotide, oxidized form. O2: oxygen. H2O: water. Cyt c: cytochrome c. CoQ10: coenzyme Q10.

Coenzyme Q10 (CoQ10) is a fat-soluble lipophilic molecule ubiquitously situated in the hydrophobic domain of all cell membranes. It particularly acts as an electron and proton carrier of the mitochondrial respiratory chain, being situated in the inner mitochondrial membrane and taking part in ATP synthesis (Figure 1) [1].

The role of CoQ10 in oxygen metabolism makes it a source of the superoxide anion radical, one of the main reactive oxygen species (ROS). In contrast, it also acts as an antioxidant by directly scavenging free radicals, protecting cell membranes from lipid peroxidation, and enhancing the activity of antioxidant enzymes, among others [1].

**Figure 2:**
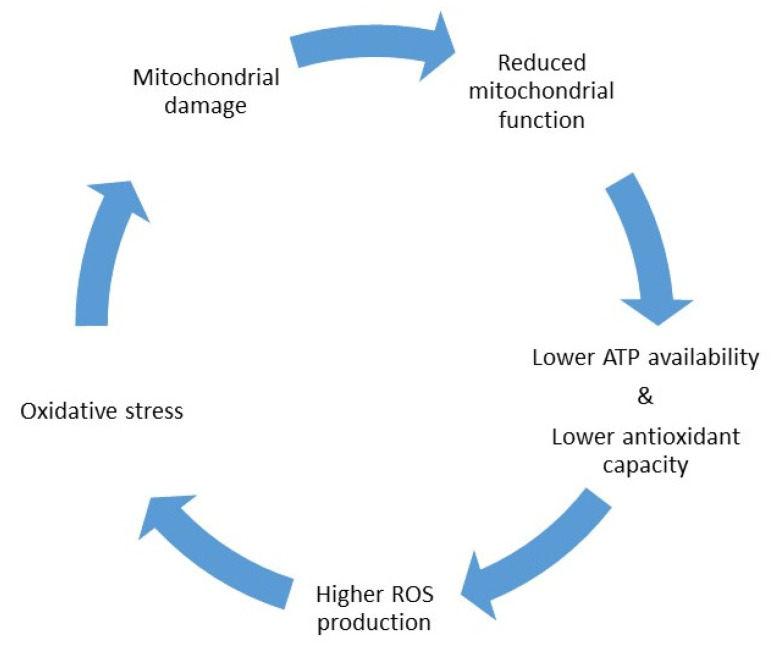
Vicious cycle between mitochondrial dysfunction and oxidative stress damage.

The dual nature of CoQ10 as a pro-oxidant and antioxidant makes it a key regulatory element of the oxidative state balance in the cell. Therefore, insufficient CoQ10 levels could lead to diminished mitochondrial respiration activity, which may result in lower ATP production, less ROS counteraction, increased oxidative stress, mitochondrial damage, and subsequent mitochondrial dysfunction. Indeed, this shapes a positive feedback system, in which lower mitochondrial activity may lead to increased oxidative stress damage, which subsequently induces mitochondrial impairment and, thus, affects the activity of these organelles (Figure 2). Therefore, oxidative stress can be caused by, or be the cause of, mitochondrial dysfunction [2], and insufficient CoQ10 levels may contribute to generate them both.

CoQ10 is endogenously synthesized in all human tissues [3]. However, insufficient levels of this molecule are associated with the consumption of some drugs [4], certain diseases in which a mutation in a gene implicated in CoQ10 synthesis is involved [5], and advanced age [6]. In the reproductive field, the decline in human oocyte quality associated with the aging process has been linked with increased oxidative stress and/or mitochondrial dysfunction [7] as mitochondria are necessary for proper meiotic spindle assembly, the segregation of chromosomes, maturation, fertilization, and embryo development [8]. Thus, insufficient CoQ10 levels may constitute a feasible explanation for this age-related oocyte quality deterioration.

Poor oocyte quality is not restricted to the aging process, as many intrinsic and external factors, including environmental pollutants [9,10], may lead to an altered microenvironment surrounding the oocyte and, thus, triggering this condition.

Oocyte maturation is achieved in follicular fluid (FF), where bidirectional communication occurs between cumulus cells and the oocyte. Thus, FF characteristics may influence the final oocyte quality [11]. In 2011, Turi’s group analyzed, for the first time, the CoQ10 levels in the FF of women undergoing infertility treatment. Even though they found no direct association between these levels and oocyte/embryo quality [12], in 2017, Akarsu et al. described better embryo morphokinetic parameters, as well as higher pregnancy rates, in women aged under 41 years with higher CoQ10 levels in FF, regardless of their age [13]. Therefore, CoQ10 deficiency, or any other cause that indirectly lowers its levels, may influence oocyte quality and could cause women’s infertility.

Finally, an altered ovarian environment with high oxidative stress damage and mitochondrial dysfunction may not be directly related to insufficient CoQ10 levels. Notwithstanding, raising CoQ10 levels may benefit oocyte quality by means of mitochondrial function enhancement and ROS counteraction.

**Figure 3:**
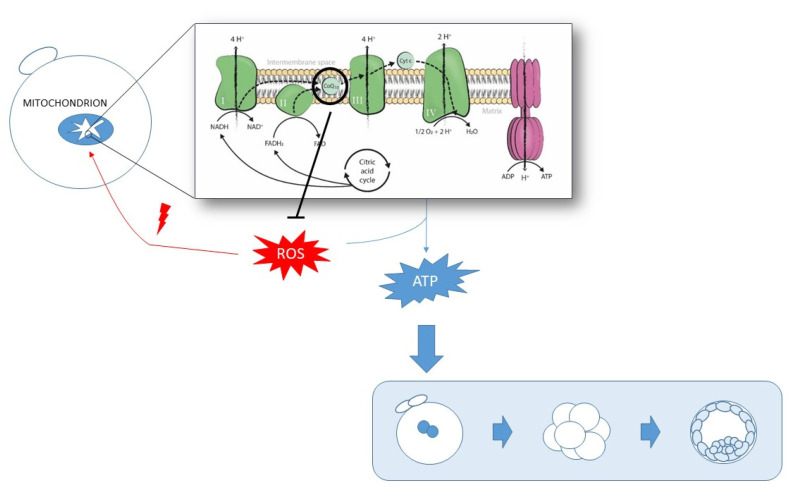
Schematic representation of the role of mitochondria and CoQ10 in acquiring optimal oocyte quality by means of ATP production and ROS counteraction.

CoQ10 supplementation was shown to be partly effective in treating many human diseases associated with mitochondrial dysfunction [14]. Increasing CoQ10 levels may reduce oxidative stress and enhance mitochondrial function to, thus, improve these patients’ symptoms. In reproduction, CoQ10 supplementation may constitute a potential therapeutic option to overcome suboptimal oocyte quality as regards the crucial role of mitochondria in achieving optimal oocyte maturity (Figure 3). This review describes the main advances to date achieved by CoQ10 supplementation in the reproductive field to improve human oocyte quality during in vitro fertilization (IVF) treatments.

This review has been made after an in-depth search in PubMed of all the scientific papers related to this topic. Keywords used for searching the studies were: “human oocyte quality”, “CoQ10”, “antioxidant”, “oxidative stress”, “mitochondrial function”, “pregnancy”, “supplementation”, and “ROS”, among others.

## 2. CoQ10 Supplementation in IVF Treatments

**Figure 4:**
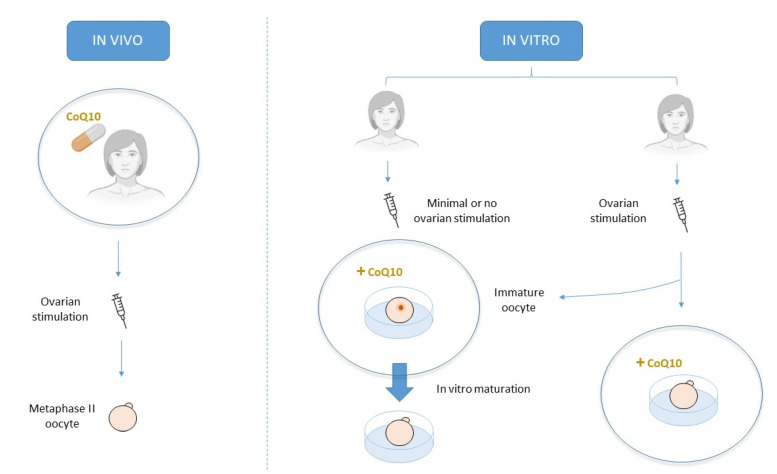
Schematic description of the main three approaches for CoQ10 treatment.

CoQ10 can be orally supplied prior to any assisted reproduction technique (ART) or as a culture media adjuvant during IVF treatment. Oral treatment attempts to improve oocyte quality in vivo, while culture media supplementation attempts to do so in vitro [2] (Figure 4).

Oral preovulatory CoQ10 treatment exerts positive effects on the ovaries of aged mice. On the one hand, it improves the ovarian reserve, ovarian response, and oocyte quality while taking oocyte mitochondrial parameters back to normal levels of young controls [15]. On the other hand, it increases the number of cumulus cells surrounding the oocyte, as well as their mitochondrial activity, which favors oocyte competence acquisition and subsequent reproductive performance [16]. Furthermore, CoQ10 addition to culture media has successfully reverted the age-induced effects observed in aged oocytes from mice [17] and pigs [18].

Increased oxidative stress damage is not only restricted to the aging process. Along these lines, CoQ10 has also proven to partially revert oxidative stress damage in the oocytes of young mice [19] and other animal models [20,21].

Finally, CoQ10 supplementation has been tested in in vitro maturation (IVM) culture. Maside et al. did not find any benefits in a porcine model [22]; however, Abdulhasan et al. reported higher maturation rates and greater mitochondrial mass and function in immature bovine oocytes after 24 h in IVM medium supplemented with 40 µM of CoQ10 [23]. Moreover, immature mouse oocytes matured in vitro and co-cultured with FF from infertile women with endometriosis demonstrated a trend toward higher maturation rates after CoQ10 supplementation compared to the very low maturity reported in the control group as a result of the endometriotic environment [24].

Human studies are analyzed in the next sections. Table 1 briefly summarizes the main discussed studies.

### 2.1. Oral Supplementation

CoQ10 oral supplementation has been studied as an ART pre-treatment in infertile patients. However, drawing conclusions about its effectiveness is still complicated mainly due to the high heterogeneity of the study population characteristics and CoQ10 treatment dose/duration as well as the primary endpoints in all the studies found in the literature. Indeed although a recent systematic review and meta-analysis of five randomized controlled trials (RCTs) concluded that CoQ10 oral supplementation increased clinical pregnancy rates (CPR) compared to a placebo or no treatment {28.8% vs. 14.1%; odds ratio (OR) 2.44, 95% confidence interval (CI) 1.30–4.59, *p* = 0.006} [36], these results lose relevance given the high heterogeneity in the analyzed RCTs. For this reason, studies are evaluated individually in the next paragraphs in an attempt to only draw conclusions based on each study’s characteristics.

As indicated earlier, plasma levels of CoQ10 lower and oocyte quality becomes impaired with advancing age [1,7]. In 2014, Bentov et al. conducted a study to evaluate if CoQ10 supplementation (600 mg/day for 2 months and up to the day of oocyte retrieval) in infertile women of advanced age (35–43 years old) was able to reduce the aneuploidy rate usually associated with oocytes from patients in this age range. 

Their results showed higher rates of top quality embryos after 48 h (81.39 ± 7.4% vs. 66.05 ± 8.6%, *p* > 0.05) and 72 h (64.74 ± 10.2% vs. 41.99 ± 9.79%, *p* > 0.05), and a lower oocyte aneuploidy rate (46.44 ± 13.9% vs. 62.77 ± 9.3%, *p* > 0.05) in the CoQ10 group vs. the control group, respectively. Although optimistic, these comparisons were not statistically significant, likely because the total sample size was not recruited. The study finished prematurely because of several concerns about the possible deleterious effects of polar body biopsy on embryo quality and implantation [25].

In 2016, Caballero et al. evaluated the effects of CoQ10 supplementation (1200 mg/day for 12 weeks) on a group of women aged between 36 and 40 years with a previous IVF cycle and poor ovarian response. They did not find any significant difference between the treatment and control groups in terms of the number of recovered mature oocytes (1.82 ± 0.82 vs. 1.87 ± 0.76 in the control group, *p* = 0.77), implantation rate (26.2% vs. 21.4% in the control group, *p* = 0.75), and clinical pregnancy rate (15.4% vs. 12.8% in the control group, *p* = 0.64) [26]. Nevertheless, fertilization, blastocyst formation, and embryo quality rates are better indicators of CoQ10 effects on oocyte quality, as implantation and pregnancy rates largely depend on the quality of the embryo chosen for transfer.

Despite the lack of hopeful CoQ10 treatment results for oocyte quality, Gat et al., in 2016, evaluated not only the oocyte and embryological parameters but also the follicular parameters in women with decreased ovarian reserve undergoing intrauterine insemination (IUI) and IVF cycles. Although no range was delimited in this retrospective analysis, the mean age recorded was around 40 years old. In the IUI cycles, CoQ10 supplementation (600 mg/day for more than 1 month) yielded a higher antral follicle count (7.4 ± 5.7 vs. 5.9 ± 4.7, *p* = 0.0001) and more mature follicles >16 mm (3.3 ± 2.3 vs. 2.9 ± 2.2, *p* = 0.01) before and after ovarian stimulation, respectively. 

However, the pregnancy rates between groups were similar. In the IVF cycles, the CoQ10 treatment group yielded a higher antral follicle count (8.6 ± 6.3 vs. 5.2 ± 5, *p* = 0.0001) but a similar number of mature follicles >16 mm (5.9 ± 4.6 vs. 5.5 ± 3.5, *p* > 0.05). Interestingly, the CoQ10 group required a significantly lower gonadotropins total dose (3414 ± 1141 vs. 3877 ± 1143, *p* = 0.003). However, in line with the findings of Bentov and Caballero, the numbers of retrieved oocytes (8.4 ± 7.1 vs. 8.5 ± 5.6), zygotes (4.7 ± 4.5 vs. 5.3 ± 4.1), and blastocysts (1.1 ± 0.8 vs. 1.4 ± 1.1), as well as pregnancy rates, were similar (*p* > 0.05) between the treatment and control groups, respectively [27]. The main limitation of this study is that it lacked a negative control group, as both groups received dehydroepiandrosterone (DHEA) supplementation based on their history of poor ovarian reserve and/or response to stimulation.

CoQ10 oral supplementation may exert a positive effect at the follicular level, although the oocytes retrieved from these “more suitable” follicles did not show any measurable upgrading. However, the study population of these three studies consisted of women of advanced age.

In contrast, CoQ10 supplementation (600 mg/day for 2 months before ovarian stimulation) increased the ovarian response {mean (interquartile range, IQR); 4 (2,5) vs. 2 (1,4) oocytes; *p* = 0.002}, fertilization rates (67.5% vs. 45.1%; *p* = 0.001) and the number of high-quality embryos {1 (0,2) vs. 0 (0, 1.75); *p* = 0.03} in young women (<35 years old) with poor ovarian reserve. The cumulative clinical pregnancy and live birth rates were similar between groups, which suggests that the larger number of high-quality embryos achieved after CoQ10 treatment was not translated to more chances of pregnancy after consecutive transfers [28].

Hence, CoQ10 supplementation prior to ovarian stimulation for ART treatment may improve the ovarian response to stimulation in aged patients by possibly creating a more suitable follicular environment. Giannubilo et al. proved that the patients receiving CoQ10 oral supplementation showed higher, but non-significant, FF CoQ10 levels in those follicles containing an oocyte compared to empty follicles (0.25 ± 0.14 vs. 0.18 ± 0.08 µg/mL; *p* = 0.058) [29]. However, this follicular improvement may not suffice to enhance oocyte quality in aged patients because it may be too late to recover from such age-related deterioration, which may lead to higher aneuploidy rates. This hypothesis is supported by the fact that CoQ10 does enhance oocyte and embryological parameters in young poor responders [28], who might have problems responding to ovarian stimulation but may still be in time to revert the oxidative stress-related damage to oocytes.

Finally, two studies conducted in clomiphene-citrate (CC)-resistant women with polycystic ovarian syndrome (PCOS) also confirmed the positive effects of CoQ10 supplementation in the follicular environment. These women are characterized by large numbers of immature follicles and the usual inability to ovulate. Hence, an improvement in the ovarian response to stimulation may better their overall success rates. 

On the one hand, CoQ10 supplementation (180 mg/day from cycle day 2 to the ovulation induction day) as an adjuvant in the CC stimulation protocol significantly increased the number of follicles >14 mm (1.94 ± 0.25 vs. 0.13 ± 0.29, *p* < 0.05) and ≥18 mm (1.85 ± 0.27 vs. 1.30 ± 0.32, *p* < 0.001) compared to a control group with only CC. This larger number of mature follicles might have led to more mature oocytes of optimal quality. Unfortunately, this was not the primary endpoint of the study and patients underwent programmed intercourses. Moreover, the ovulation rate was significantly higher in the CoQ10 group (65.9% vs. 15.5%, *p* < 0.001), as were the serum estradiol and progesterone levels (168.93 ± 75.01 vs. 138.32 ± 70.24 pg/mL, *p* < 0.05, and 10.2 ± 1.03 vs. 8.9 ± 0.91, *p* < 0.001, respectively) [30]. 

On the other hand, CoQ10 supplementation (180 mg/day from cycle day 1) as an adjuvant in the CC and gonadotrophin stimulation protocol significantly increased the size of the mature follicle (9.4 vs. 7.8 mm in the control group, *p* < 0.05) [31].

### 2.2. Culture Media Supplementation

Alternatively, CoQ10 can be added directly to in vitro culture media, which comes into close contact with the oocyte. In this manner, and unlike oral supplementation, this treatment does not focus on improving ovarian response to stimulation because it may attempt to improve oocyte quality in a more direct way. This can be done by adding CoQ10 to either standard culture media, in which already mature oocytes have been placed, or in vitro maturation media, in which immature oocytes are found.

#### 2.2.1. Standard Culture

CoQ10 supplementation in standard culture media aims to improve the quality of the mature oocytes retrieved after an ovulation trigger. Therefore, these oocytes have already undergone meiosis I in vivo and are arrested in the metaphase of the second meiosis when exposed to CoQ10.

The first meiosis has been related to the origin of a high percentage of aneuploidies in human embryos, which are associated mainly with advanced age [37]. Hence, exposing the oocytes in metaphase II to CoQ10 could be too late to counteract age-related oxidative stress-related damage.

Indeed Kile et al. conducted a prospective clinical trial in sibling embryos from women of advanced age (>35 years old). After ICSI, 1/3 of the zygotes from the same oocyte cohort were cultured in media supplemented with mitoTEMPO and Mitoquinol, two mitochondria-targeted antioxidants, while the remaining zygotes were cultured in standard media with no supplement to act as a control group. 

There were no significant differences between the study and control groups, respectively, regarding the fertilization rates (91% vs. 83%, *p* = 0.11), D5 (20% vs. 18%) or total (45% vs. 48%) good quality blastocyst development (per zygote), or in total blastocyst development (62% vs. 63%) or euploidy rates (30 vs. 33%). Even though this study did not evaluate CoQ10 supplementation at the oocyte level, it proved the safety of this treatment in in vitro culture, as well as its inefficiency in improving aged women’s embryo quality, whose oocytes and subsequent embryos were already affected by the aging process [32].

MitoTEMPO and Mitoquinol are two examples of mitochondria-targeted antioxidants [38,39]. These therapeutics are now being increasingly studied for their proven higher potential for scavenging mitochondrial ROS. Unlike untargeted general antioxidants, these molecules are able to cross the mitochondrial bilayer and accumulate in the mitochondrial matrix and, thus, act at the ROS production site [39]. Mitoquinol is the reduced form of mitoquinone mesylate (MitoQ) [40], a ubiquinone moiety of the CoQ10 molecule bound to a highly cationic carrier that preferentially accumulates in mitochondria [39]. This molecule is further discussed below.

#### 2.2.2. In Vitro Maturation Culture

CoQ10 supplementation to standard culture media may not overcome the oxidative stress-related damage that affects the first meiosis course. A mild ovarian stimulation protocol, or even no stimulation at all, followed by IVM of the retrieved immature oocytes, may solve this problem. By this technique, oocytes are retrieved before the first meiosis is completed, and thus CoQ10 supplementation to culture media at this point may improve their oxidative status, enhance the mitochondrial function, and, subsequently, better their overall quality. Alternatively, immature oocytes deriving from conventional stimulation cycles, which have received ovulation trigger in vivo but have not completed meiosis I when retrieved, may benefit from this treatment [41].

Ma et al. recently published a study in which immature oocytes, obtained after ovarian stimulation and from small follicles of ≤12 mm, were randomly assigned to IVM culture with or without CoQ10. After being retrieved, immature oocytes were cultured in IVM medium with or without 50 µM of CoQ10 for 24–48 h before undergoing a polar body biopsy to evaluate their chromosomal status. Women were classified into two groups according to their age: young group (≤30 years old) and older group (≥38 years old). 

In the young group, there were no significant differences in maturation (80% vs. 76.9%, *p* = 0.748) and post-meiotic aneuploidy rates (28.6% vs. 30%, *p* = 0.905) between the CoQ10 and the control group, respectively. In the older group, maturation and post-meiotic aneuploidy rates were significantly higher (82.6% vs. 63%, *p* = 0.035) and lower (36.8% vs. 65.5%, *p* = 0.02), respectively, in the CoQ10 group [34]. In addition, the mitochondrial mass was significantly higher in the CoQ10 group after a Mitotracker analysis [33].

As mentioned in the previous section, MitoQ constitutes another available and promising CoQ10 supplementation approach. MitoQ treatment (50 nM) has been found to enhance maturation rates (77% vs. 51%; *p* < 0.05) and mitochondrial membrane potential, as well as to lower the percentage of oocytes with misaligned chromosomes (25% vs. 61%; *p* < 0.05) in human immature oocytes deriving from stimulated cycles compared to a control group. Interestingly, the women recruited in this study were aged between 29 and 45 years, and the first two listed parameters similarly improved across age groups [35].

These findings suggest that both CoQ10 and MitoQ may be able to improve the mitochondrial function of oocytes that have already undergone an ovulation trigger in vivo, but failed to achieve the final maturation status prior to follicular aspiration. However, while both seemed to improve the oocyte quality of older women [34,35], only MitoQ displayed significant differences regardless of the patient’s age [35].

## 3. Discussion

CoQ10 has been shown to be a safe and well-tolerated antioxidant treatment in humans [1]. Some adverse effects, such as nausea, diarrhea, and abdominal pain, have been described after CoQ10 intake in the treatment of other diseases [42,43]. However, they are mild and occasionally-occurring side effects [44].

It is also a versatile therapy as it can be administered following a wide variety of protocols and at different ART treatment time points. Oral CoQ10 may benefit women with poor ovarian reserve, poor response to ovarian stimulation, advanced age, or PCOS. What these all have in common are fewer and, usually less competent, mature oocytes [45,46]. However, promising results have been found mostly in follicular terms [27,30,31], and an enhancement at the oocyte level was achieved only in a population of young poor responders [28]. This finding suggests that the lower age-related CoQ10 levels might be too low to be rescued after this antioxidant treatment. These patients may need higher doses or a different administration protocol, which have not yet been defined.

Regarding the beneficial effects of CoQ10 supplementation at the follicular level, higher levels of this molecule may create a more favorable environment for developing competent follicles. It was shown that oxidative stress leads to higher apoptotic processes in granulosa cells [47]. CoQ10, by means of counteracting oxidative stress, can reduce this programmed granulosa cell death and, thus, reduce follicular atresia. This is evidenced by the higher antral follicle counts and larger number of mature follicles recorded in some reviewed studies [27,30]. 

However, this improvement did not suffice to significantly enhance oocyte quality, which has been directly evaluated in only a few studies [25,27], but is indirectly evidenced by similar pregnancy outcomes in others [26,27,28]. It is important to bear in mind that, although CoQ10 can have an impact at the follicular level, the ultimate objective of every ART treatment is to achieve a successful pregnancy, which means that clear upgrades in pregnancy rates are needed to introduce this treatment into routine clinical practice.

Another approach is to supplement CoQ10 directly in vitro during IVF treatment. High levels of this antioxidant come into close contact with the oocyte, although its apparent positive action at the follicular level is absent. In this context, CoQ10 supplementation does not offer any advantage over the standard culture of fertilized oocytes from women of advanced age [32], which seems logical if we consider that these oocytes had already undergone two consecutive meiotic divisions with age-related damaged cell machinery. 

For this reason, CoQ10 supplementation during the IVM of immature aged oocytes, which are arrested in the prophase of the first meiosis, appears more plausible. Indeed, promising results were shown in this line [34], which suggest that CoQ10 might help these aged oocytes to properly resume meiosis, as evidenced by lower aneuploidy rates. CoQ10 might achieve this by improving the mitochondrial function [48], as evidenced by the increased mitochondrial mass in treated oocytes [33] and, thus, provide the energy they lack due to the aging process, which is essential for acquiring final maturation. 

In any case, the improvement was not fully achieved as more age-related factors contribute to this poor oocyte quality [49], and CoQ10 treatment itself may not be sufficient to overcome them. In contrast, CoQ10 addition during IVM of oocytes from young women did not show any advantage [34], which suggests that these oocytes already had the sufficient energy needed to resume meiosis, and higher CoQ10 levels did not lead to any advantage. Thus, other strategies to improve maturation rates in such patients should be investigated.

Nevertheless, MitoQ supplementation during IVM culture showed significant improved oocyte quality regardless of patients’ age [35]. We hypothesize that the advantageous location of this targeted molecule and its ability to concentrate at higher rates in mitochondria may favor its mechanism of action and, thus, exert significant changes on young oocytes. MitoQ (or any other mitochondria-targeted antioxidant) supplementation deserves further research in human clinical trials.

In any case, the majority of the studies herein discussed focused on clinical outcomes, and did not evaluate the effects of CoQ10 on the oxidative stress status or at the mitochondrial level in oocytes. Ma et al. in 2018 and Al-Zubaidi et al. in 2021 were the only ones to analyze such parameters, and they proved higher mitochondrial mass and mitochondrial membrane potential, respectively, after CoQ10/MitoQ addition to IVM medium [34,35]. However, they did not evaluate oxidative stress markers or any other indicator of oocyte energy status as many animal studies have previously done [15,18,19,23].

Therefore, further research is needed in this field, and should focus mainly on the molecular level to understand the exact mechanism by which CoQ10 enhances mitochondrial function. By solving this research question, we would be able to establish the best protocol, dose, molecular form, and approach for its administration. Presently, our recommendation is to continue investigating this antioxidant in the reproductive field, however, mostly as an oral treatment or during IVM. Its addition to fertilized oocytes during standard culture appears worthless as its main role in improving oocyte competence should be performed prior to completing second meiosis and likely even earlier. In addition, more attention should be paid to mitochondria-targeted antioxidants, which have been poorly studied in human clinical trials and appear to be more efficient than the isolated CoQ10 form.

## 4. Conclusions

CoQ10 constitutes a safe, well-tolerated therapy capable of improving oocyte quality through oxidative stress counteraction and mitochondrial function enhancement. In humans, oral CoQ10 supplementation appears to exert positive effects, particularly at the follicular level, by creating a more favorable environment for competent follicle development. However, these benefits are not necessarily translated to substantial oocyte improvements and subsequent gestational results. Indeed, no improvement has been reported regarding the final pregnancy outcomes using this therapy. CoQ10 addition to culture media appears effective if performed in the immature stages. In this scenario, mitochondria-targeted molecules may confer a certain advantage and offer a better prognosis.

Hence, the available data reviewed in this work do not clearly prove the advantage of CoQ10 supplementation in improving human oocyte quality. Thus, this still promising avenue deserves further research, particularly using these modified CoQ10 forms, as well as with molecular studies evaluating the impact of this therapy on the oxidative stress status and mitochondrial function in human gametes.

## Figures and Tables

**Table 1:** Summary of the main human studies evaluating CoQ10 supplementation both in vivo and in vitro.

		Study	Design	Treatment Groups (*n*)	Population	Outcomes	Results
**In vivo**		Bentov et al., 2014 [25]	RCT	CoQ10 gr.: 17 Control gr: 22	IVF patients 35–43 y.o.	% Top quality embryos	X
						% Aneuploidy	X
		Caballero et al., 2016 [26]	RCT	CoQ10 gr.: 39 Control gr.: 39	Poor ovarian responders 36–40 y.o.	No. mature oocytes	X
						Pregnancy outcomes	X
		Gat et al., 2016 [27]	Retrospective	IUI cycles CoQ10 gr.: 330 Control gr.: 467	Poor ovarian reserve IUI patients	Antral follicle count	☺
						No. follicles >16 mm	☺
						Pregnancy outcome	X
				IVF cycles CoQ10 gr.: 78 Control gr.: 175	Poor ovarian reserve IVF patients	Antral follicle count	☺
						No. follicles >16 mm	X
						No. oocytes	X
						No. zygotes	X
						No. blastocysts	X
						Pregnancy outcome	X
		Xu et al., 2018 [28]	RCT	CoQ10 gr.: 76 Control gr.: 93	Poor ovarian responders <35 y.o.	No. oocytes	☺
						% Fertilization	☺
						No. Top quality embryos	☺
						Pregnancy outcome	X
		Giannubilo et al., 2018 [29]	Prospective	15	IVF patients 31-46 y.o. with CoQ10 treatment	FF CoQ10 levels in follicles with an oocyte	X
		El Refaeey et al., 2014 [30]	RCT	CoQ10 gr.: 51 Control gr.: 50	CC-resistant PCOS	No. follicles >14 mm and ≥18 mm	☺
						% Ovulation	☺
						E2 and P4 levels	☺
		Sen Sharma et al., 2017 [31]	RCT	CoQ10 gr.: 32 Control gr.: 30	CC-resistant PCOS	Mature follicle size	☺
**In vitro**	Standard Culture	Kile et al., 2020 [32]	RCT	CoQ10 gr.: 66 Control gr.: 143 zygotes	IVF patients 35–46 y.o.	% Fertilization	X
						% Top quality embryos	X
						% Euploidy	X
	IVM	Ma et al., 2018 [33]	RCT	CoQ10 gr.: 32 Control gr.: 32 immature oocytes	IVM patients 35–46 y.o.	Mitochondrial mass	☺
		Ma et al., 2020 [34]	RCT	Women ≤30 y.o. CoQ10 gr.: 37 Control gr.: 37 immature oocytes from stimulated cycles	IVF patients ≤30 y.o.	% Maturation	X
						% Post-meiotic oocyte aneuploidy	X
				Women ≥38 y.o. CoQ10 gr.: 46 Control gr.: 46 immature oocytes from stimulated cycles	IVF patients ≥38 y.o.	% Maturation	☺
						% Post-meiotic oocyte aneuploidy	☺
		Al-Zubaidi et al., 2021 [35]	RCT	MitoQ gr.: 44 Control gr.: 45 immature oocytes from stimulated cycles	IVF patients 29–45 y.o.	% Maturation	☺
						Mitochondrial membrane potential	☺
						% Oocytes with misaligned chromosomes	☺

A smiley face emoji means significant and positive improvement after CoQ10 treatment vs. the control group, while a cross denotes that CoQ10 treatment did not offer any clear benefit. Gr.: group. RCT: randomized controlled trial. y.o.: years old. IUI: intrauterine insemination. IVF: in vitro fertilization. CC: clomiphene-citrate. PCOS: polycystic ovarian syndrome. FF: follicular fluid. E2: estradiol. P4: progesterone.
